# Peritumoral Neuropilin-1 and VEGF receptor-2 expression increases time to recurrence in hepatocellular carcinoma patients undergoing curative hepatectomy

**DOI:** 10.18632/oncotarget.2553

**Published:** 2014-10-11

**Authors:** Peng-Yuan Zhuang, Jian-Dong Wang, Zhao-Hui Tang, Xue-Ping Zhou, Yong Yang, Zhi-Wei Quan, Ying-Bin Liu, Jun Shen

**Affiliations:** ^1^ Department of General Surgery, Xinhua Hospital, School of Medicine, Shanghai Jiao Tong University, Shanghai, 200082, China

**Keywords:** Neuropilin-1, vascular endothelial growth factor receptor-2, peritumoral tissue, overall survival, recurrence

## Abstract

**Purpose:**

To determined Neuropilin-1 (NRP-1) and vascular endothelial growth factor receptor 2 (VEGFR-2) expression in the tumoral and peritumoral tissues of 214 treatment-naïve HCC patients and its correlation with overall survival (OS) and time to recurrence (TTR).

**Experimental Design:**

NRP-1 and VEGFR-2 expression were examined by tissue microarray and peritumoral hypoxia by pimonidazole staining and angiogenesis by microvessel density (MVD). OS and TTR were evaluated by Kaplan-Meier analysis and log-rank test.

**Results:**

Peritumoral NRP-1 and VEGFR-2 expression were significantly higher than that of the tumoral tissue (*p* < 0.001 for both), and high peritumoral expression of both factors was negatively associated with tumor size (*p* < 0.001 for both). Patients with high peritumoral expression of both proteins had the longest median OS (>94.0 months) and TTR (>84.0 months). The multivariate Cox proportional hazards analysis revealed that patients with high peritumoral expression of both NRP-1 and VEGFR-2 were more than 4 times less likely to have recurrence (*p* = 0.004) and more than 10 times likely to survive (*p* < 0.001).

**Conclusions:**

Peritumoral NRP-1 and VEGFR-2 expression is associated with prolonged TTR and extended OS of HCC patients and both may be useful as predictors of surgical outcome of HCC patients and explored as potential therapeutic targets.

## INTRODUCTION

Liver cancer is the fifth most common cancer globally and the second most frequent cause of cancer mortality worldwide with approximately 750,000 new cases annually [1, 2]. Hepatocellular carcinoma (HCC) is the most common type of liver cancer and half of the new cases are in China [3]. Although significant improvement in diagnosis and treatment of HCC has been achieved, the outcome of patients with advanced HCC remain dismal with a median survival of merely a few months, mandating diagnosing the disease at early stage for an imporved outcome [4]. High intrahepatic recurrence rate post curative hepatectomy is also a major problem [5]. Furthermore, biomarkers that are currently used clinically to predict the prognosis of HCC patients after curative surgical resection remain unsatisfactory in terms of both accuracy and reproducibility [6]. Therefore, it remains clinically important to identify novel prognostic biomarkers to improve the diagnosis and treatment of HCC patients.

Neuropilin-1 (NRP-1), which was first described as a semaphoring receptor important for the guidance of developing neurons [7, 8], is expressed on endothelial cells and acts as a co-receptor for vascular endothelial growth factor receptor 2 (VEGFR-2)/VEGF-A, thereby being implicated in VEGF-A mediated angiogenesis and vasculogenesis [9]. Recent studies have also revealed an important role of NRP-1 malignant progression of many cancers [10]. Bergé *et al.* showed that increased NRP1 expression in human tumor hepatocytes was significantly associated with primary HCC and blocking NRP-1 function inhibited vascular remodeling and tumor xenograft growth in mice [11]. However, its role and its correlation with VEGFR-2 in HCC remain largely unknown.

HCC is a vascular tumor that proliferates through angiogenic pathways mediated, in part, by VEGFR-2 [12]. Previous studies have demonstrated that tumoral angiogenesis involving VEGF-A and its two receptors, VEGFR1/flt-1 and VEGFR-2/KDR, is associated with the prognosis of HCC patients [13–14]. Currently, information on angiogenesis and biomarkers has been obtained mostly from tumor tissue, while scant information is available from peritumoral tissue. The microenvironment of the peritumoral liver tissue such as the inflammation or angiogenesis status may be a favorable soil for the spread of HCC cells. It has been reported that higher contents of certain pro-angiogenetic factors were found in the peritumoral liver tissue than the tumor tissue [15–18]. Budhu *et al.* found that intrahepatic venous metastasis was associated with a unique immune/inflammation response signature in the peritumoral liver tissue but not in the intratumoral microenvironment [19], indicating that the peritumoral liver tissue may impact on the prognosis and intrahepatic metastasis of HCC. Although NRP-1 and VEGFR-2 are expressed on endothelial cells and tumor cells [20], their expression in the corresponding peritumoral tissues has not been examined [21, 22], especially in the peritumoral liver tissue of HCC patients.

We hypothesized that peritumoral NRP-1 and VEGFR-2 expression in HCC patients may differ from that in the tumoral tissue and may be associated with the surgical outcome. In the present study, we investigated the expression of NRP-1 and VEGFR-2 in the tumoral and peritumoral tissues by tissue microarrays and immunohistochemistry from 214 treatment-naïve HCC patients who had received curative hepatectomy at our institution and analyzed whether their expression correlated with the overall survival (OS) and time to recurrence (TTR). We also investigated whether peritumoral NRP-1 and VEGFR-2 expression correlated with peritumoral hypoxia in human tissue specimens and in mouse xenografts bearing human HCC cells.

## PATIENTS AND METHODS

### Patients

We prospectively recruited 968 consecutive patients with pathologically proven HCC who underwent curative resection between January, 2004 and December, 2011 at the Department of Surgery, Jiaotong University, and 214 patients were randomly retrieved from our database. None of the patients received any preoperative anticancer treatment. HCC was staged according to the UICC TNM classification system (7th Edition) and tumor differentiation was graded by the Edmondson-Steiner grading system. The Scheuer system was applied for grading inflammatory activity and staging fibrosis and cirrhosis of the peritumoral liver tissue [23, 24].

### Tissue microarray and immunohistochemistry

We constructed tumor microarray (TMA) (Shanghai Biochip Co., Ltd, Shanghai, China), and 2 cores were taken from representative formalin-fixed paraffin-embedded tumor tissue and liver tissue adjacent to the tumor within a distance of 10 mm to construct TMA slides. Duplicate cylinders from two different areas, a total of four punches from each patient were obtained. Immunohistochemistry was performed by a two-step method using the Envision-plus detection system (Dako, Glostrup, Denmark). The following primary antibodies were used: mouse monoclonal anti-NRP-1 antibody and anti-VEGFR-2 antibody (both from Santa Cruz Biotechnology, Santa Cruz, CA), rabbit polyclonal anti-CD31 antibody and anti-HIF-1α antibody (both from Abcam, Cambridge, MA). The measurement of the density of positive staining was conducted by Integrated optical density (IOD) which was determined using Image-Pro Plus v6.2 software (Media Cybernetics, Inc., Bethesda, MD) with the same setting for all the slides; For determination of the high or low expression, optimal cutoff values were estimated by X-tile software v3.6.1 (Yale University, New Haven, CT) to distinguish patients with poor or good prognosis to achieve a lowest p value and a highest hazard ratio [25], therefore, in our study, the 75th percentile values (IOD, 857757.19) were used to define high and low peritumoral expression of NRP-1, and the median value (IOD, 527462.13) were used to define high and low peritumoral expression of VEGFR-2. For quantification of mean microvessel density (MVD), five fields at × 100 magnification in CD31 stained “hotspot” were captured and MVD was quantified as CD31-positive area/total area.

### Quantitative real-time RT-PCR

Total cellular RNA was extracted using Trizol reagents following the manufacturer's protocol (Invitrogen, Carlsbad, CA), and RNA was reverse transcribed using Primescript™ RT (TaKaRa, Otsu, Shiga, Japan). Quantitative real-time PCR was performed using a SYBR Premix Ex *Taq*™ (TaKaRa). The following primers used in our study were shown in Supplementary Table 1. The relative amount of tissue mRNA, standardized by the amount of β-actin mRNA, was expressed as −ΔCT = [CT (factor) –CT (β-actin)]. The ratio of the number of mRNA copies to the number of β-actin mRNA copies was then calculated as 2^−ΔCT^×K, where K is a constant.

### Tumor xenograft

Six week old male BALB/c nu/nu nude mice, weighing approximately 20g each (Shanghai Institute of Materia Medica, Chinese Academy of Sciences, Shanghai, China), were housed in laminar flow cabinets under specific pathogen-free conditions. Orthotopic implantation of human HCC cells HCCLM3 in the BALB/c mice was carried out as previously described [26–28].

### Cells and cell culture

Human hepatic cell line L02 was obtained from the Shanghai Institute of Cell Biology, Chinese Academy of Sciences (Shanghai, China) [29]. The cells were maintained in Dulbecco-modified Eagle medium (DMEM) containing 10% fetal calf serum, 100 U/mL penicillin and 50 g/mL streptomycin. Cells were incubated in 37°C under a humidified atmosphere containing 5% CO_2_. For growing under hypoxia, L02 cells were incubated with cobalt chloride (100 mol/L) for 24 hours.

### Immunocytochemistry staining

Primary antibodies were a rabbitmonoclonal NRP-1 antibody (1:200, Abcam, Cambridge, MA), a mouse monoclonal VEGFR-2 (1:50, Abcam, Cambridge, MA). Primary antibodies were detected by using secondary antibodies of anti-rabbit IgG-TR(Texas Red) (Abcam, Cambridge, MA), anti-mouseIgG-FITC (Abcam, Cambridge, MA), respectively. L02 were cultured on attachment factor-coated slide wells (Sonic Seal Slide Well, Nalge Nunc International). Cells were incubated with primary antibodies overnight at 4°C. Sections were washed three times in PBS, followed by secondary antibody for 1h at room temperature. Samples were analyzed with an inverted fluorescence microscope (Olympus IX51) equipped with an Olympus Qcolor 3 digital camera (Olympus).

### Pimonidazole staining for hypoxia

Mice bearing HCCLM3 xenografts were injected intravenously with pimonidazole hydrochloride at 60 mg/kg (Chemicon International, Temecula, CA) 90 minutes before euthanasia. The xenografts were then removed and immediately fixed in 10% buffered formalin in the dark. Slides (4mm thick) with peritumoral tissue preparations were incubated with polyclonal rabbit antiserum to pimonidazole-protein adducts, which were visualized with 3, 3-diaminobenzidine. Area fractions showing pimonidazole staining were determined by image analysis.

### Follow-up

Patients were followed up by clinic visit every 2 months during the first postoperative year and at least every 3–4 months thereafter. A CT scan of the chest and abdomen was performed every 6 months. Bone scanning or magnetic resonance imaging (MRI) was done if localized bone pain was reported. If recurrence was suspected, CT scan or MRI was performed immediately.

### Statistical analysis

Data were analyzed using the SPSS 15.0 for Windows (SPSS Inc., Chicago, IL). Spearman rank correlation coefficient determination was used to analyze correlation among parameters. OS or TTR was defined as the interval between surgery and death of any cause or recurrence; if recurrence was not diagnosed during this study, the cases were censored on the date of death or the last date of follow-up. Early recurrence was defined using 1 year as the cutoff value of TTR, as suggested by Poon *et al*'s study [30]. Kaplan-Meier analysis and log-rank test were used to compare OS and TTR. *p* < 0.05 was considered statistically significant.

## RESULTS

### Patient demographic and baseline characteristics

A total of 968 consecutive patients with pathologically proven HCC received curative resection at our institution during the review period, and 214 patients were randomly retrieved from our database. None of the patients received any preoperative anticancer treatment. Patient demographic and baseline characteristics were shown in Supplementary Table 2. Most patients were male and positive for HBsAg and had hepatic cirrhosis. The mean tumor size was 6.6±4.0cm. The majority had TNM stage I HCC and showed grade I or II tumor differentiation. Lymph node metastasis was present in less than 5% of the patients.

### Expression of NRP-1 and VEGFR-2 in the tumoral and peritumoral tissue

**Figure 1:**
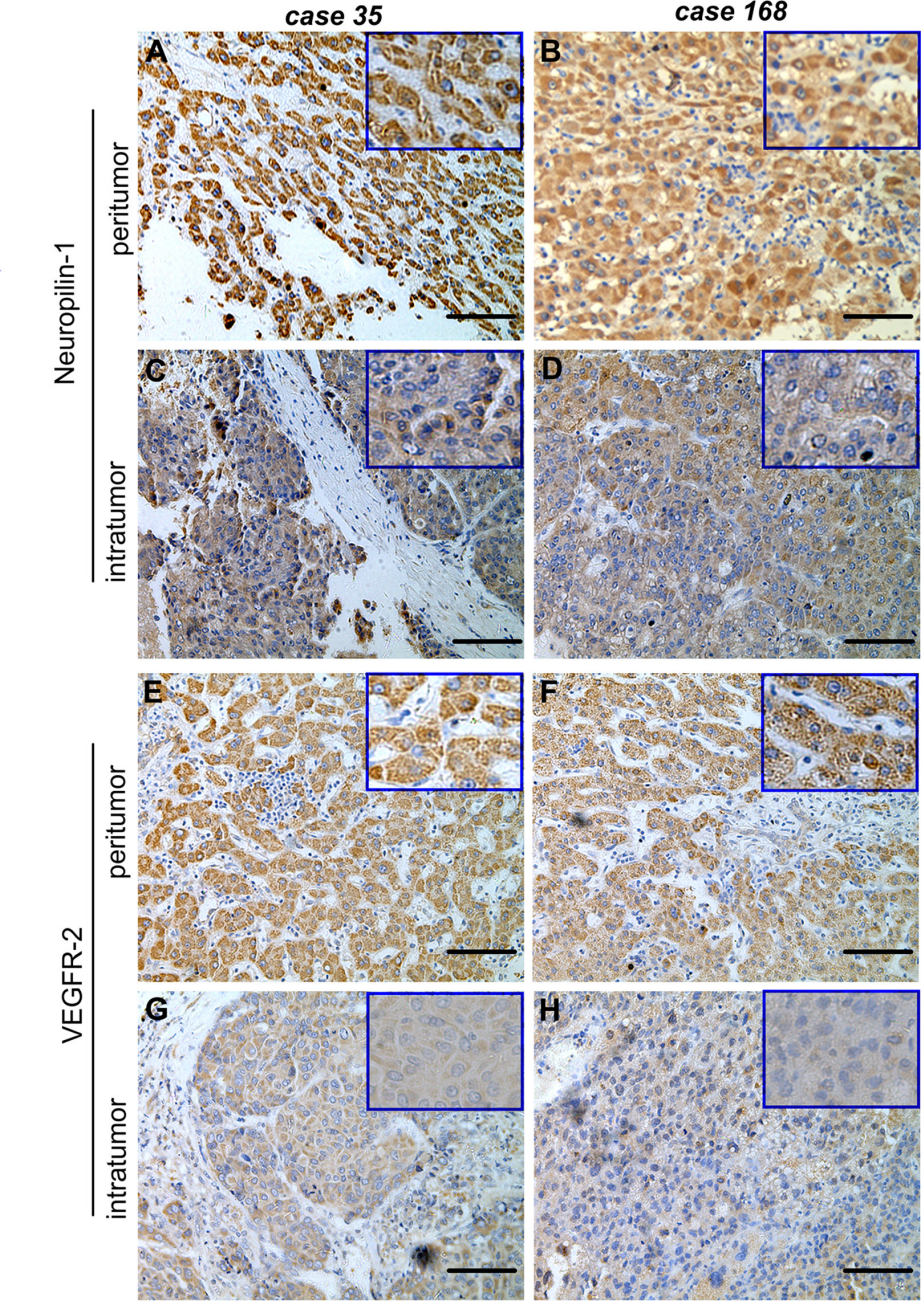
Peritumoral NRP-1 and VEGFR-2 expression were significantly higher than that of the tumoral tissue (**A** to **D**) Neuropilin-1 (NRP-1) and (**E** to **H**) vascular endothelial growth factor receptor-2 (VEGFR-2) are differentially expressed in the tumor tissue and peritumoral tissue from hepatocellular carcinoma (HCC) patients. Tumoral and peritumoral tissue specimens were obtained from patients with pathologically confirmed HCC and NRP-1 in the peritumoral (A, B) and tumoral tissue (C, D) and VEGFR-2 in the peritumoral (E, F) and tumoral tissue (G, H) were examined by tissue microarray and immunohistochemistry. Panels from representative patients are shown. Higher density of staining is seen for both NRP-1 and VEGFR-2 in the peritumoral tissue vs. the tumoral tissue. Bar, 50um.

We examined the expression of VEGFR-2 and NPR-1 in the tumoral and peritumoral tissue specimens of HCC patients by tissue microarray and immunohistochemistry. VEGFR-2 and NPR-1 were mostly detected in the hepatocytes and weakly in stromal cells in the peritumoral tissues. The content of NRP-1 in the tumor tissues (308787.75±27988.79) was significantly lower than that of the peritumoral tissue (726480.76±22573.14; *p* < 0.001) (Fig. 1A to 1D). The level of VEGFR-2 in the tumor tissues (24248.06±7710.75) was also markedly lower than that of the peritumoral tissue (598084.29±23245.87; *p* < 0.001) (Fig. 1E to 1H).

We found peritumoral NRP-1 and VEGFR-2 correlated significantly (cc = 0.648, *p* = 0.004); Using the 75th percentile value of peritumoral NRP-1 and the median value of peritumoral VEGFR-2 as the cutoff for high and low expression of the two proteins, 74.8% of the patients exhibited low expression of NRP-1 and 50.5% had low expression of VEGFR-2. Compared to patients who had high peritumoral expression of NRP-1, patients with low peritumoral NRP-1 expression had a markedly larger tumor size (*p* = 0.019), a greater number of satellite lesions (*p* = 0.009), and a higher proportion of grade 3 and 4 inflammatory activity (*p* = 0.003) and stage 4 hepatic cirrhosis of peritumoral liver tissue (*p* = 0.001) (Table 1). Additionally, compared to those with high peritumoral VEGFR-2 expression, patients with low peritumoral VEGFR-2 expression also had a markedly larger tumor size (*p* = 0.019) and a greater number of satellite lesions (*p* = 0.009). Spearman correlation analysis further showed that high peritumoral NRP-1 or VEGFR-2 expression was negatively associated with tumor size (NRP-1: r = −0.588, *p* < 0.001; VEGFR-2: r = −0.455, *p* < 0.001). We further validated the tissue microarray findings in an independent cohort of 69 HCC patients (Supplementary Table 3) by quantitative RT-PCR assays. We found that the mRNA transcript levels of *NRP-1* were −11.6±0.25 in the tumor tissues and −2.3±0.15 in the peritumoral liver tissues (*p* < 0.001). Additionally, the mRNA transcript levels of *VEGFR-2* were −13.5±0.12 in the tumor tissues, which were significantly lower than those of peritumoral liver tissues (−3.3±0.25; *p* < 0.001). These findings suggest that NRP-1 and VEGFR-2 are differentially expressed in the tumor and peritumoral tissues of HCC patients and high NRP-1 or VEGFR-2 expression are associated a smaller tumor size and fewer satellite lesions.

**Table 1:** Patient demographic and baseline characteristics stratified by peritumoral NRP-1 and VEGFR-2 expression

	Low NRP-1	High NRP-1	*P*	Low VEGFR-2	High VEGFR-2	*P*
No. (%)	160 (74.8)	54 (25.2)		108 (50.5)	106 (49.5)	
Age, years† mean (SD)	51.4 (12.2)	49.5 (12.6)	0.232	52.3 (12.5)	50.4 (13.5)	0.154
Male gender, n (%)	145 (90.6)	49 (90.7)	0.980	98 (90.7)	96 (90.6)	0.965
HBsAg positivity, n (%)	128 (80.0)	48 (88.9)	0.139	92 (85.2)	84 (79.2)	0.256
ALT (U/L)†	43.8 (23.6)	37.1 (38.2)	0.237	45.3 (38.3)	44.6 (32.9)	0.778
AFP (ng/mL)†	4750.61 (1088.11)	3938.49 (1453.80)	0.353	4164.35 (1006.80)	4699.92 (1629.73)	0.740
Tumor size (cm)†	7.12 (3.56)	5.64 (4.19)	0.019	7.21 (4.51)	5.31 (4.37)	0.003
Satellite lesion, n (%)	38 (23.8)	4 (7.4)	0.009	32 (29.6)	10 (9.4)	0.000
Vascular invasion, n (%)	68 (42.5)	20 (37.0)	0.481	50 (46.3)	38 (35.8)	0.120
Lymph node metastasis, n (%)	8 (5.0)	2 (3.7)	0.742	6 (5.6)	4 (3.8)	0.748
TNM stage, IIIA, n (%)	38 (23.8)	8 (14.8)	0.227	28 (25.9)	18 (17.0)	0.279
Edmondson grade, III–IV, n (%)	44 (27.5)	12 (22.2)	0.445	30 (27.8)	26 (24.5)	0.589
**Scheuer's score**						
Grade 3-4	78 (48.8)	14 (25.9)	0.003	50 (46.3)	42 (39.6)	0.324
Stage (cirrhosis) 4	134 (83.8)	34 (63.0)	0.001	88 (81.5)	80 (75.5)	0.285
Peritumoral HIF-1α†	54453.06 (7710.75)	23081.12 (5454.23)	0.003	65567.12 (5125.24)	31752.02 (6520.34)	0.002
MVD in tumor†	7.78% (0.40%)	3.22% (0.23%)	0.012	8.39% (0.45%)	3.08% (0.20%)	0.002
MVD in peritumoral tissue†	2.78% (0.06%)	0.43% (0.02%)	0.024	3.25% (0.02%)	0.67% (0.03%)	0.005

AFP, alpha fetoprotein; ALT, alanine transaminase; MVD, microvessel density.

† Student's *t* test, and the results were expressed as mean (SD)

### High peritumoral NRP-1 and VEGFR-2 expression is associated with marked improvement in time to recurrence and overall survival

The patients were followed up for a mean duration of 20.2 (range, 1.9 to 94.0) months and at the last follow-up visit, 80 (37.4%) patients experienced tumor recurrence, and 58 (27.1%) patients died, including 18 patients who died of liver failure without confirmed tumor recurrence. The 1-, 3- and 5-year OS rates were 84%, 58% and 44%, respectively. Using tumor recurrence at one year as the cutoff for early tumor recurrence [30], patients with high peritumoral NRP-1 expression had a markedly lower incidence of early recurrence (11.1%, 6/54 vs. low NRP-1 expression: 26.9%, 43/160, *p* = 0.017). Moreover, patients with high peritumoral VEGFR-2 expression also had a significantly lower incidence of early recurrence (5.7%, 6/106 vs. low VEGFR-2 expression: 43.5%, 47/108, *p* < 0.001).

**Figure 2:**
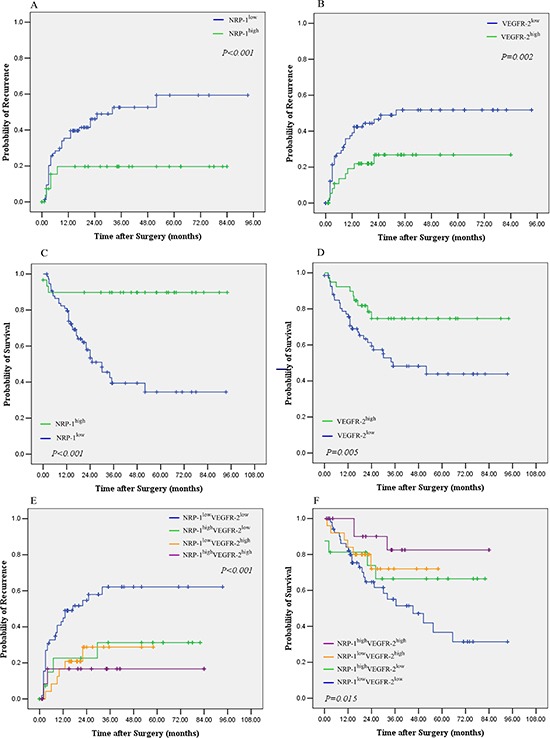
Kaplan–Meier curves of overall survival (OS) and time to recurrence (TTR) of patients with high or low peritumoral NRP-1 and VEGFR-2 expression **(A)** TTR stratified by high vs. low NRP-1 expression; **(B)** TTR stratified by high vs. low VEGFR-2 expression; **(C)** OS stratified by high vs. low NRP-1 expression; **(D)** OS stratified by high vs. low VEFGR-2 expression; **(E)** TTR stratified by both NRP-1 and VEGFR-2; **(F)** OS stratified by both NRP-1 and VEGFR-2.

**Figure 3:**
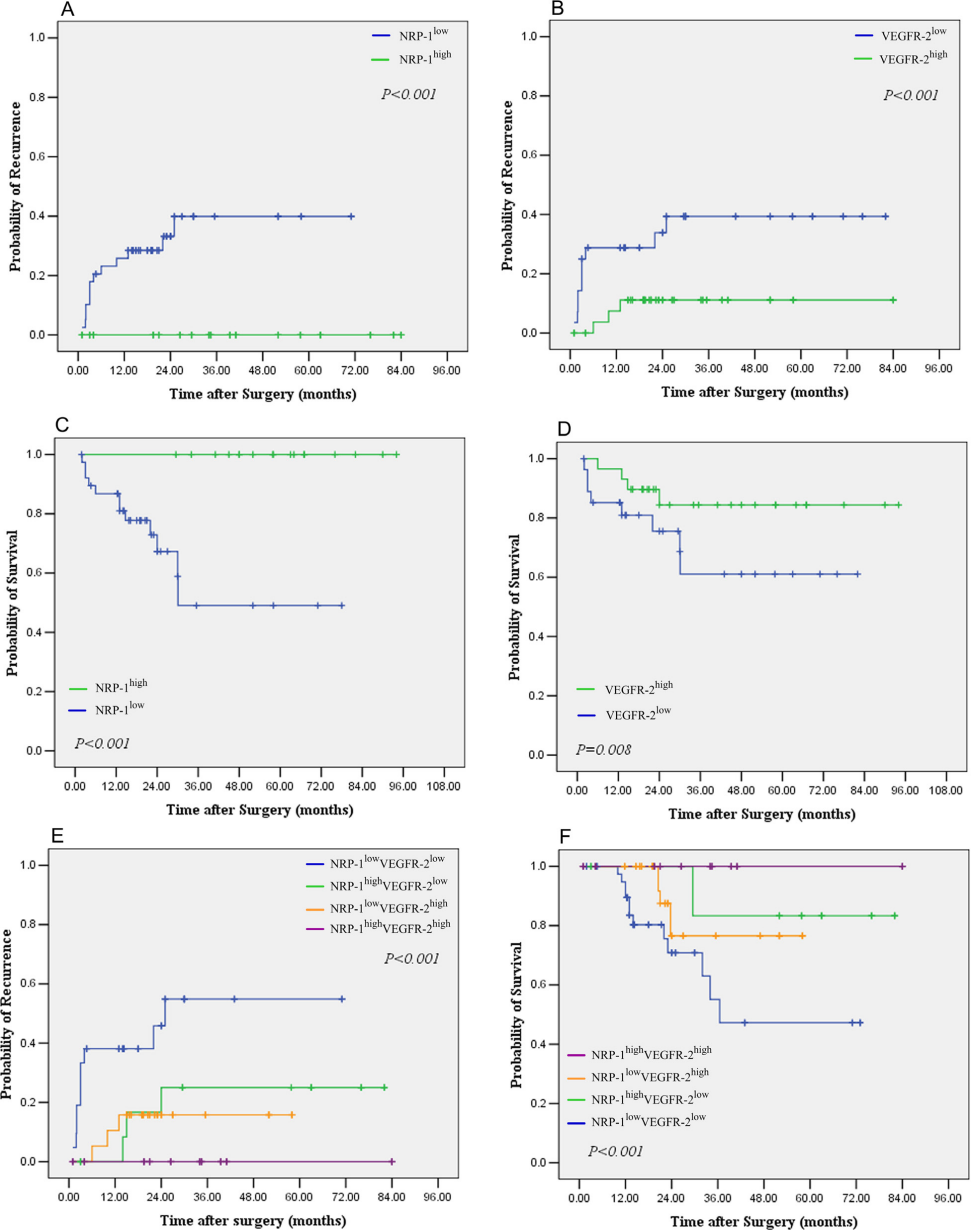
Kaplan–Meier curves of overall survival (OS) and time to recurrence (TTR) of patients with high or low peritumoral NRP-1 and VEGFR-2 expression with a maximum tumor diameter of 5 cm (n = 88) **(A)** TTR stratified by high vs. low NRP-1 expression; **(B)** TTR stratified by high vs. low VEGFR-2 expression; **(C)** OS stratified by high vs. low NRP-1 expression; **(D)** OS stratified by high vs. low VEFGR-2 expression; **(E)** TTR stratified by both NRP-1 and VEGFR-2; **(F)** OS stratified by both NRP-1 and VEGFR-2.

The median TTR was >84.0 months for patients with high peritumoral NRP-1 expression and was significantly longer than that of patients with low peritumoral NRP-1 expression (22.5 months, *p* < 0.001) (Fig. 2A). The median TTR for patients with high peritumoral VEGFR-2 expression was >84.0 months, which was markedly longer than that of patients with low peritumoral VEGFR-2 expression (21.8 months, *p* = 0.002) (Fig. 2B). The median OS was >94.0 months for patients with high peritumoral NRP-1 expression and was significantly longer than that of patients with low peritumoral NRP-1 expression (33.2 months, *p* < 0.001) (Fig. 2C). The median OS for patients with high peritumoral VEGFR-2 expression was >94.0 months, which was significantly longer than that of patients with low peritumoral VEGFR-2 expression (34.4 months, *p* = 0.005) (Fig. 2D). Moreover, patients with low peritumoral expression of both NRP-1 and VEGFR-2 had the shortest median OS (45.3 months) and TTR (18.9 months) and patients with high peritumoral expression of both NRP-1 and VEGFR-2 had the longest median OS (>94.0 months) and TTR (>84.0 months) (Fig. 2E to 2F). When tumor size was excluded as a determinant of outcome, high peritumoral NRP-1 or VEGFR-2 expression was still associated with markedly longer OS and TTR in patients with a maximum tumor diameter of 5 cm (Fig. 3A-3F).

Our univariate analysis revealed that NRP-1 and VEGFR-2 expression in the peritumoral tissues was associated with TTR (*p* < 0.001 for NRP-1 and *p* = 0.002 for VEGFR-2) and OS (*p* < 0.001 for NRP-1 and *p* = 0.005 for VEGFR-2) (Supplementary Table 4). Risk factors (LNM, satellite lesions, tumor size, cancerous thrombi, cirrhosis nodules) identified by univariate analysis were pooled into a multivariate Cox proportional hazards analysis (Table 2 and Supplementary Table 5), which further showed that patients with high peritumoral NRP-1 expression were approximately 3 fold less likely to have recurrence (HR: 0.334; 95% CI: 0.130−0.513; *p* = 0.012) (Table 2). Moreover, patients with high peritumoral VEGFR-2 expression were more than twice less likely to have recurrence (HR: 0.433; 95% CI: 0.206–0.878; *p* = 0.015). Furthermore, patients with high peritumoral expression of both NRP-1 and VEGFR-2 were more than 4 times less likely to have recurrence (HR: 0.218; 95%CI: 0.114–0.488; *p* = 0.004). Patients with high peritumoral NRP-1 expression were more than 7 fold more likely to survive (HR: 0.129; 95% CI: 0.039−0.426; *p* = 0.001) (Table 2). Moreover, patients with high peritumoral VEGFR-2 expression were more than twice likely to survive (HR: 0.410; 95% CI: 0.195-0.863; *p* = 0.005). Importantly, patients with high peritumoral expression of both NRP-1 and VEGFR-2 were more than 10 times likely to survive (HR: 0.098; 95%CI: 0.024−0.217; *p* < 0.001). (Table 2).

**Table 2:** Multivariate analyses of correlation of high peritumoral NRP-1 and VEGFR-2 expression with time to tumor recurrence (TTR) and overall survival (OS)

	HR	95% CI	*P*
**TTR**			
High peritumoral NRP-1	0.334	0.130–0.513	0.012
High peritumoral VEGFR-2	0.433	0.206–0.878	0.015
High peritumoral NRP-1 and VEGFR-2	0.218	0.114–0.488	0.004
**OS**			
High peritumoral NRP-1	0.129	0.039–0.426	0.001
High peritumoral VEGFR-2	0.410	0.195–0.863	0.005
High peritumoral NRP-1 and VEGFR-2	0.098	0.024–0.217	< 0.001

### Peritumoral NRP-1 and VEGFR-2 expression is associated with tumoral and peritumoral hypoxia

We further examined whether peritumoral NRP-1 and VEGFR-2 expression was associated with hypoxia in the tumoral and peritumoral tissues of HCC patients by immunohistochemistry. We found that peritumoral HIF-1α content was 23081.12±5454.23 in patients with high peritumoral NRP-1 expression, which was markedly lower than that of patients with low peritumoral NRP-1 expression (54453.06±7710.75, *p* = 0.003) (Table 1). Moreover, peritumoral HIF-1α content was 31752.02±6520.34 in patients with high peritumoral VEGFR-2 expression, which was significantly lower than that of patients with low peritumoral VEGFR-2 expression(65567.12±5125.24, *p* = 0.002). Furthermore, patients with high peritumoral NRP-1 expression had a markedly lower MVD in the tumor tissue (3.22%±0.23% vs. low peritumoral NRP-1: 7.78%±0.40%; *p* = 0.012) and in the peritumoral tissue (0.43%±0.02% vs. low peritumoral NRP-1: 2.78%±0.06%; *p* = 0.024). Patients with high peritumoral VEGFR-2 expression had a markedly lower MVD in the tumor tissue (3.08%±0.20% vs. low peritumoral VEGFR-2: 8.39%±0.45%; *p* = 0.002) and in the peritumoral tissue (0.67%±0.03% vs. low peritumoral VEGFR-2: 3.25%±0.02%; *p* = 0.005). Moreover, patients with low peritumoral expression of both NRP-1 and VEGFR-2had the highest MVD (tumor: 9.05%±0.12%; peritumoral tissue: 4.16%±0.08%) while patients with high peritumoral expression of both NRP-1 and VEGFR-2 had the lowest MVD (tumor: 2.20%±0.02%; pritumoral tissue: 0.33%±0.01%; both were *p* < 0.001).

**Figure 4:**
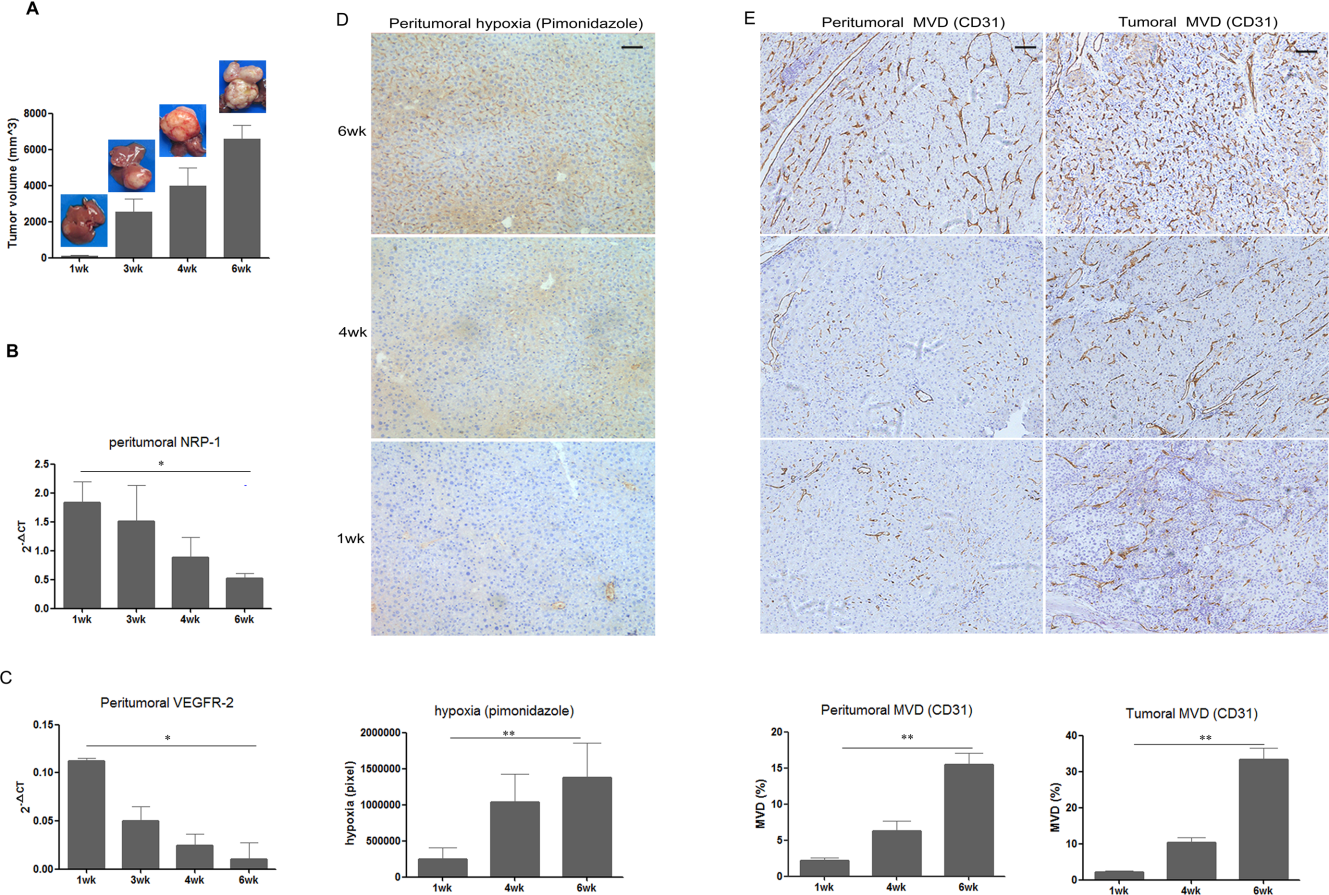
Peritumoral NRP-1 and VEGFR-2 expression, hypoxia level and MVD, as well as tumoral MVD in the 6wk of tumor growth in a mouse orthotopic xenograft model bearing human HCC-LM3 cells **(A)** Growth of mouse xenografts bearing human HCC cells LM3. Expression of NRP-1 **(B)** and VEGFR-2 **(C)** in the peritumoral hepatic tissue of mice bearing human HCC xenografts was examined by real-time quantitative RT-PCR and normalized against β-actin. **(D)** Pimonidazole staining for peritumoral hypoxia. **(E)** Tumoral and peritumoral microvessel density (MVD) in the mouse xenograft was determined as described in Methods. Data in **(B)** to **(E)** are expressed as mean ± SD of at least three independent experiments. *p < 0.05; **p < 0.01; Bar, 50um.

We then examined the expression of NRP-1 and VEGFR-2 in the peritumoral tissue in a mouse orthotopic xenograft model bearing human HCC-LM3 cells (Fig. 4A). As expected, our quantitative RT-PCR assays using primers specific for human *NRP-1* and *VEGFR-2* failed to detect both *NRP-1* and *VEGFR-2* in the peritumoral tissue while RT-PCR assays using primers specific for mouse *NRP-1* and *VEGFR-2* showed a time dependent decrease in both *NRP-1*(*p* = 0.013) and *VEGFR-2* (*p* = 0.023)in the peritumoral tissue (Fig. 4B and 4C), indicating that tumor growth impacted on the expression of *NRP-1* and *VEGFR-2* in the peritumoral hepatic tissue. Pimonidazole staining of mouse xenograft showed increasing hypoxia of the peritumoral tissues over time (*p* = 0.007, Fig. 4D) and immunohistochemical staining further revealed increasing MVD in the tumoral (*p* = 0.002) and peritumoral tissue (*p* = 0.002) over time as tumor xenografts grew (Fig. 4E).

**Figure 5:**
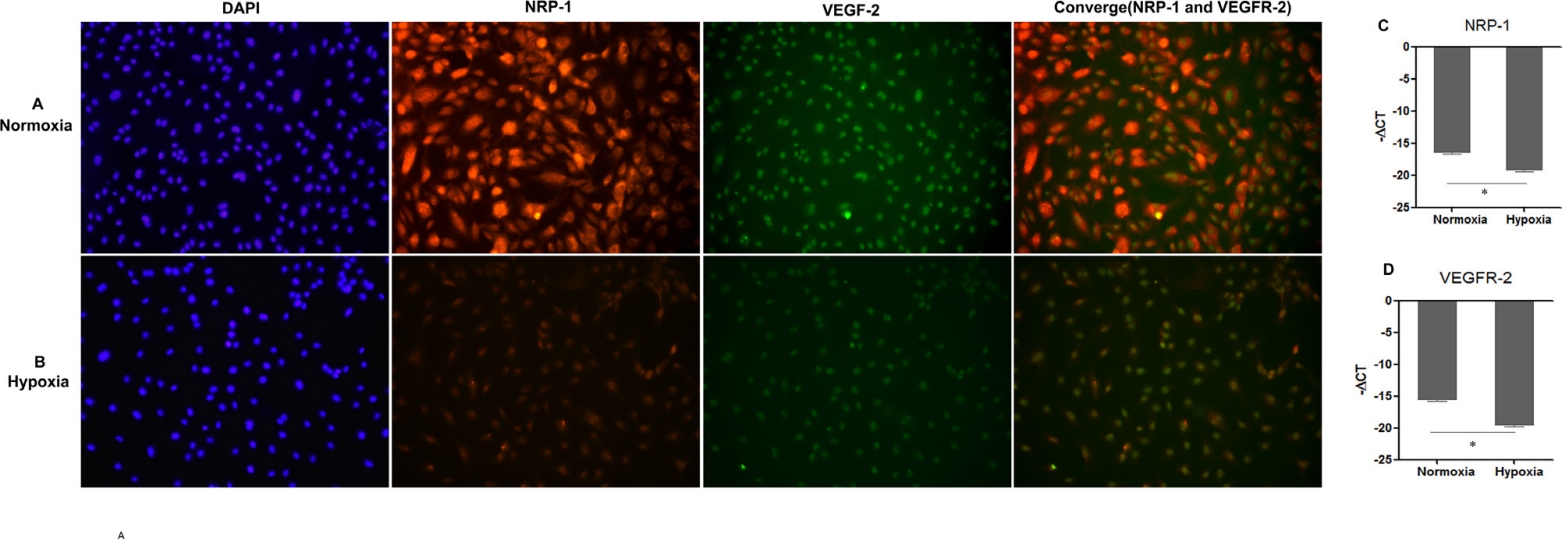
Hypoxia suppressed the expression of NRP-1 and VEGFR-2 in hepatic cells L02 Panels for representative NRP-1 and VEGFR-2 expression from cobalt chloride-induced hypoxia condition **(A)** and normoxia condition **(B)** were shown by immunocytochemistry staining. The mRNA transcript levels of NRP-1**(C)** and VEGFR-2 **(D)** were determined by real-time quantitative RT-PCR and normalized against β-actin. Data are expressed as mean ± SD of at least three independent experiments. *p < 0.05

Our RT-PCR assays and pearson correlation analysis additionally demonstrated that peritumoral NRP-1 and VEGFR-2 expression was negatively correlated with peritumoral HIF-1α (NRP-1: r = −0.512, *p* < 0.001; VEGFR-2: r = −0.603, *p* < 0.001) and tumoral (NRP-1: r = −0.365, *p* = 0.006; VEGFR-2: r = −0.622, *p* < 0.001) and peritumoral MVD (NRP-1: r = −0.712, *p* < 0.001; VEGFR-2: r = −0.574, *p* < 0.001). Cobalt chloride-induced hypoxia of hepatic L02 cells markedly reduced NRP-1 and VEGFR-2 expression confirmed by the immunocytochemistry staining (Fig 5A to B), and the decreased mRNA transcript levels of *NRP-1* (−19.2±0.23 vs. normoxia: −16.4±0.22, *p* = 0.018, Fig 5C) and *VEGFR-2* (−19.5±0.27 vs. normoxia −15.6±0.23, *p* = 0.022, Fig 5D) was also confirmed by the RT-PCR.

## DISCUSSION

The current study demonstrated that NRP-1 and VEGFR-2 were expressed in peritumoral liver tissue, mainly in the hepatocyte, at a markedly higher level than that in tumor tissue. More importantly, we provide the initial clinical evidence that high peritumoral NRP-1 and VEGFR-2 expression is associated with markedly longer OS and TTR of HCC patients. Furthermore, our multivariate Cox proportional hazards analysis showed that patients with high peritumoral expression of both NRP-1 and VEGFR-2 were more than 10 times likely to survive. These findings strongly indicate that peritumoral NRP-1 and VEGFR-2 are prognostic determinants of HCC. Our findings differ from those by Chen *et al.* who found that high NRP-1 levels were associated with an adverse prognosis for bladder cancer patients [31], but are consisten twith those by Younan *et al.* who found that higher NRP-1 levels correlated with complete remission of acute myeloid leukemia and acute lymphoblastic leukemia patients [32], suggesting that the effect of NRP-1 may be cancer type specific. It is also worthy of note that the studies by Chen *et al.* and Younan *et al.* only examined tumoral NRP-1 content.

Because of the intimate relation between VEGFR-2 in angiogenesis and the recent findings implicating NRP-1 in VEGFR-2 mediated angiogenesis [6, 8], we examined peritumoral NRP-1 and VEGFR-2 expression and peritumoral hypoxia. We found that peritumoral NRP-1 and VEGFR-2 expression was negatively correlated with peritumoral hypoxia and tumoral and peritumoral MVD. The findings are further corroborated by our mouse xenograft studies. Tumor hypoxia is known to impact on the outcome of HCC. Yang *et al.* recently showed that high expression of HIF-1α in HCC tissues was closely associated with a lower OS and disease-free survival of HCC patients [33]. Similarly, Osada *et al.* demonstrated that positive HIF-1α expression was associated with poor prognosis of ovarian carcinoma patients [34]. Although our present study did not find the direct effect on prognosis exerted by peritumoral hypoxia, however, peritumoral hypoxia was negatively correlated with low peritumoral NRP-1 and VEGFR-2 expression which was associated with poor prognosis. We showed that patients with high peritumoral expression of both NRP-1 and VEGFR-2 had the lowest MVD. We further found that high peritumoral NRP-1 and VEGFR-2 expression also correlated with smaller primary tumor size and fewer satellite lesions. Our mouse xenograft studies further showed that the expression of both NRP-1 and VEGFR-2 significantly declined as the tumor xenograft grew. To our knowledge, these findings provide the first piece of evidence implicating peritumoral NRP-1 and VEGFR-2 in tumor growth, angiogenesis and development of satellite lesions.

The specific functions of NRP-1 in vessel development and angiogenesis are not yet fully known. Previous studies have shown that NRP-1 acts as a co-receptor for VEGFR-2/VEGFA, and NRP-1 mediates a VEGFA/NRP-1/VEGFR-2 pathway, leading to tumor angiogenesis [20]. However, little is known about the role of NRP-1 and VEGFR-2 in peritumoral liver tissue. We postulate that the same combination of VEGFA/NRP-1/VEGFR-2 or VEGFA/NRP-1 or VEGFA/VEGFR-2 is also present in hepatocytes, which renders the peritumoral hepatocytes as the storage site for VEGFA, resulting in a decoy effect on competition for VEGF binding with endothelial cells. In other words, the gradient of NRP-1 and VEGFR-2 expression between the tumor and peritumoral tissue may attract VEGFA into the peritumoral tissue, where it combines with peritumoral NRP-1 and VEGFR-2 in hepatocytes, which may decrease tumor endothelial cell proliferation. On the other hand, the binding of peritumoral VEGF-A with hepatocytes through NRP-1 or VEGFR-2 may starve the endothelial cells, ultimately decreasing the tumoral and peritumoral MVD as observed in our present study. To some extent, this postulation could be further confirmed by the *in vitro* study that under the stimulation of VEGF-165 at the same dose, the effect on the the proliferation of both peritumoral and tumor endothelial cells by co-culturing of hepatocytes and the endothelial cells.

We speculate that after curative hepatectomy, peritumoral hepatocytes expressing abundant of NRP-1 or VEGFR-2 may play a positive role by providing an infertile soil for endothelial cells and primary tumor and subclinical metastatic tumor cells. Our findings demonstrate that NRP-1 and VEGFR-2 expressed by peritumoral liver cells predict a favorable postoperative outcome with prolonged TTR and extended OS of HCC patients and highlight the important role of VEGFRs expressed on residual liver cells, which may exert a decoy effect on competition for VEGFA binding with endothelial cells, providing a hostile peritumoral environment for recurrence and metastasis. Our study has significant clinical implications inhelping identify a high-risk subgroup of patients with low expression of both NRP-1 and VEGFR-2 for whom adjuvant therapies after hepatectomy are needed.

## SUPPLEMENTARY TABLES


