# Supplementary material for: Peritumoral Neuropilin-1 and VEGF receptor-2 expression increases time to recurrence in hepatocellular carcinoma patients undergoing curative hepatectomy
Source: Oncotarget. 2014 Oct 11;5(22):11121–32. doi: 10.18632/oncotarget.2553 (PMC4294350; doi:10.18632/oncotarget.2553)
Supplement: Supplementary file 1 [file oncotarget-05-11121-s001.pdf]

SUPPLEMENTARY TABLES

Supplementary Table S1. Primers used for real time-PCR

Gene name	Orientation	Primer sequence (5′ – 3′)	Species
NRP-1	forward	CACAGTGGAACAGGTGATGACTTC	human
NRP-1	reverse	AACCATATGTTGGAAACTCTGATTGT	human
VEGFR-2	forward	AAGGCGAGACCTGCATTC	human
VEGFR-2	reverse	CTGCCCTCTTCTGAGCTCT	human
β-actin	forward	CATCTCTTGCTCGAAGTCCA	human
β-actin	reverse	ATCATGTTTGAGACCTTCAACA	human
NRP-1	forward	TCAGGACCATACAGGAGATGG	mouse
NRP-1	reverse	TGACATCCCATTGTGCCAAC	mouse
VEGFR-2	forward	TACACAATTCAGAGCGATGTGTGGT	mouse
VEGFR-2	reverse	CTGGTTCCTCCAATGGGATATCTTC	mouse
β-actin	forward	CACGATGGAGGGGCCGGACTCATC	mouse
β-actin	reverse	TAAAGACCTCTATGCCAACACAGT	mouse

Supplementary Table S2. Patient demographic and baseline characteristics

Variables	
**No.**	214
**Age, years**	
Median(range)	51 (16–82)
**Male gender, n(%)**	194 (90.7)
**Preoperative ALT, U/L**	
Mean(SD)	42.5 (31.3)
**α - Fetoprotein, ng/mL**	
Mean(SD)	4432.3 (1213.7)
**Liver cirrhosis, n(%)**	
Yes	168 (78.5)
**HBsAg, n(%)**	
Positive	176 (81.9)
**Tumor size, cm**	
Mean(SD)	6.6 (4.0)
**Lymph node metastasis, n(%)**	
Yes	10 (4.7)
**Satellite lesions, n(%)**	
Yes	44 (20.6)
**Vascular invasion, n(%)**	
Yes	88 (41.1)
**Tumor differentiation, n(%)**	
I, II	158 (73.8)
III, IV	56 (26.2)
**TNM stage, n(%)**	
I	138 (64.5)
II	30 (14.0)
IIIA	46 (21.5)

Supplementary Table S3. Demographic and baseline characteristics of HCC patients for PCR assays

Variables	
**No.**	69
**Age, years**	
Median(range)	53 (25–72)
**Male gender, n(%)**	63 (91.3)
**Preoperative ALT, U/L**	
Mean(SD)	42.6 (30.8)
**α -Fetoprotein, ng/mL**	
Mean(SD)	4361.4 (1432.3)
**Liver cirrhosis, n(%)**	
Yes	54 (78.3)
**HBsAg, n(%)**	
Positive	56 (81.2)
**Tumor size, cm**	
Mean(SD)	6.7 (4.8)
**Lymph node metastasis, n(%)**	
Yes	3 (4.4)
**Satellite lesions, n(%)**	
Yes	12 (17.4)
**Vascular invasion, n(%)**	
Yes	27 (39.1)
**Tumor differentiation, n(%)**	
I, II	52 (75.4)
III, IV	17 (24.6)
**TNM stage, n(%)**	
I	50 (72.5)
II	9 (13.0)
IIIA	10 (14.5)

Supplementary Table S4. Univariate analysis of factors associated with overall survival (OS) and time to recurrence (TTR)

	OS (*P*)	TTR (*P*)
Lymph node metastasis: no vs. yes	< 0.001	0.004
Satellite lesions: no vs. yes	< 0.001	< 0.001
Tumor size (cm): ≤5 vs. >5	< 0.001	< 0.001
Cancerous thrombi: no vs. yes	< 0.001	< 0.001
Cirrhosis nodules: no vs. yes	0.011	0.055
Peritumoral NRP-1: low vs. high	< 0.001	< 0.001
Peritumoral VEGFR-2: low vs. high	0.005	0.002
Combination of peritumoral NRP-1 and VEGFR-2	0.015	< 0.001

Supplementary Table S5. Multivariate analyses of association of clinicopathologic characteristics with time to tumor recurrence (TTR) and overall survival (OS)

	HR	95% CI	*P*
**TTR**			
Lymph node metastasis	2.210	1.347–3.612	0.010
Satellite lesions	1.554	1.308–2.549	< 0.001
Tumor size	1.719	1.562–2.332	< 0.001
Cancerous thrombi	1.513	1.211–1.915	0.002
**OS**			
Lymph node metastasis	1.557	0.989–2.534	0.063
Satellite lesions	1.512	1.442–1.973	0.005
Tumor size	2.914	1.584–5.502	< 0.001
Cancerous thrombi	1.947	1.519–3.341	< 0.001
Cirrhosis nodules	2.813	0.878–4.142	0.056
